# Optimization of sequence alignment for simple sequence repeat regions

**DOI:** 10.1186/1756-0500-4-239

**Published:** 2011-07-20

**Authors:** Abdulqader Jighly, Aladdin Hamwieh, Francis C Ogbonnaya

**Affiliations:** 1 International Center for Agricultural Research in the Dry Areas (ICARDA), P.O. Box 5466, Aleppo, Syria

## Abstract

**Background:**

Microsatellites, or simple sequence repeats (SSRs), are tandemly repeated DNA sequences, including tandem copies of specific sequences no longer than six bases, that are distributed in the genome. SSR has been used as a molecular marker because it is easy to detect and is used in a range of applications, including genetic diversity, genome mapping, and marker assisted selection. It is also very mutable because of slipping in the DNA polymerase during DNA replication. This unique mutation increases the insertion/deletion (INDELs) mutation frequency to a high ratio - more than other types of molecular markers such as single nucleotide polymorphism (SNPs).

SNPs are more frequent than INDELs. Therefore, all designed algorithms for sequence alignment fit the vast majority of the genomic sequence without considering microsatellite regions, as unique sequences that require special consideration. The old algorithm is limited in its application because there are many overlaps between different repeat units which result in false evolutionary relationships.

**Findings:**

To overcome the limitation of the aligning algorithm when dealing with SSR loci, a new algorithm was developed using PERL script with a Tk graphical interface. This program is based on aligning sequences after determining the repeated units first, and the last SSR nucleotides positions. This results in a shifting process according to the inserted repeated unit type.

When studying the phylogenic relations before and after applying the new algorithm, many differences in the trees were obtained by increasing the SSR length and complexity. However, less distance between different linage had been observed after applying the new algorithm.

**Conclusions:**

The new algorithm produces better estimates for aligning SSR loci because it reflects more reliable evolutionary relations between different linages. It reduces overlapping during SSR alignment, which results in a more realistic phylogenic relationship.

## Background

Microsatellites, or simple sequence repeats (SSRs), are tandemly repeated DNA sequences with a period of from 1 to 6 base pairs [1]. It is sometimes referred to as a variable number of tandem repeats or VNTRs. An SSR which contains one type of repeats, is called a simple SSR (e.g. (CA)_15_) and those which have more than one type are called compound SSRs (e.g. (CA)_8_(CG)_12_) [2]. The repeat units are generally di-, tri- tetra- or pentanucleotides. They are commonly found in non-coding regions of the genome.

SSRs are highly mutable loci [3]. In animals, observed SSR mutation rates have been of the order of 10^-3 ^to 10^-4 ^for autosomal repeat loci [4,5] (Wiessenbach *et al*. 1992; Weber and Wong 1993). However the average of mutations in SSR loci is 10^-2 ^in one generation [6].

Chistiakov et al. [7] suggested that two mechanisms are responsible for the high mutability in SSRs. First, motif repetition makes SSRs prone to mutation by DNA polymerase slippage during replication because of the multi-complementary sequences, and second, unequal crossing over or related processes [8-11]. The slippage rate is correlated to SSR length and this makes longer SSRs more variable than shorter ones [12,13]. However, there is no threshold length for slippage mutations [14]. The mutations that happen because of the polymerase slippage could be considered as special types of insertion/deletion (INDELs) mutations that usually occur when adding or erasing sequences without any substitution. Substitution is considered as another kind of mutation called single nucleotide polymorphism (SNPs). In general, SNPs occur much more frequently than INDELs [15]. But SSR replication slippage generates more genetic change in eukaryotes than do all base substitution per generation [16], so it increases the frequency of INDELs. In addition, it has been reported that the perfect SSR motifs are significantly more variable compared to imperfect repeated motifs [17,18].

The power of SSR regions relies on their high abundance in the genome, codominant nature, extensive genome coverage, and high polymorphism [19]. The polymorphism of SSR depends on the differences in the numbers of repeated units between alleles at a single locus. The SSRs are used as molecular markers in a wide range of applications, such as genome mapping, marker assisted selection, gene tagging, and evolutionary and diversity studies [20] The main feature of SSRs that makes them amenable for use as molecular markers is that the flanking regions are highly conserved, allowing the use of specific PCR primers to amplify the same SSR even across different taxa [21,22].

Sequence alignment involves the identification of the correct location of INDELS that have happened since their divergence from a common precursor. The true alignment reflects the evolutionary relationships between the sequences accurately. Nevertheless, in the case of a compound SSR region, the general alignment will show many overlaps between the different units of repeats, which seem biologically incorrect because of the replication slippage mutations rate. This suggests a need to re-evaluate the general alignment methods and their parameters. In this paper, we surmise that correct alignment should put the repeats separately without overlapping between them and without changing the alignment parameters. We suggest the incorporation of a simple algorithm for the shifting process of SSR loci after applying the usual alignment used in regular software.

## Findings

### Algorithm

**Figure 1:**
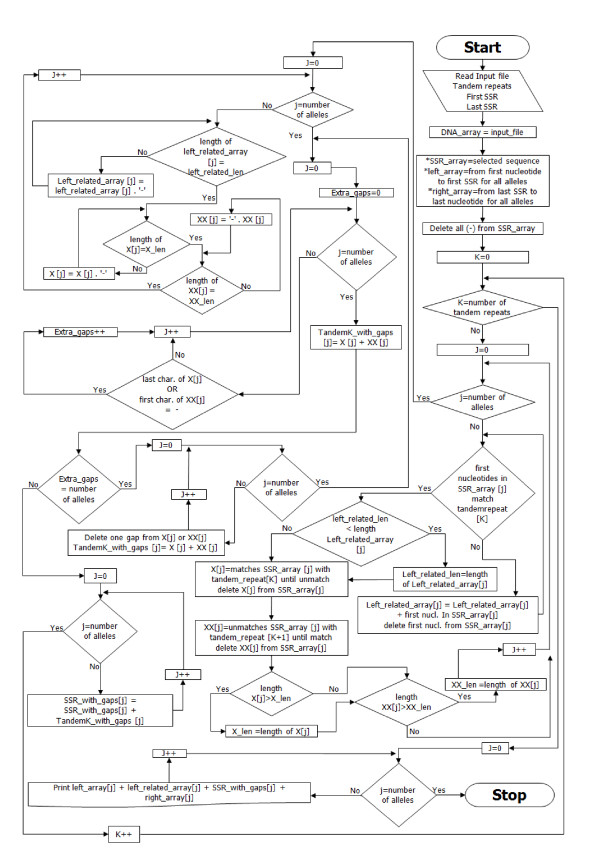
**The SSR aligning algorithm**.

In this paper, we compare our new algorithm for SSR alignment with the common alignment algorithms used in other programs. The new algorithm (Figure 1) would deal with the SSR according to the following major steps:

1- User must identify the following items:

a. Data set file

b. Repeated units

c. SSR length (first and last nucleotide)

2- Identify the sequences that do not match the first repeated unit from the beginning of the selected SSR region

3- Do this for each repeated unit

a. Put the tandem repeat in a temporary array

b. Check if the next nucleotides match the next repeated unit

c. If not, put the unmatched nucleotides in another temporary array

d. Fill the gaps to the longest sequence of the repeats in the same array

e. Merge the temporary arrays

4- Put your results instead of the SSR region.

See the additional file 1: SALT.swf. An animation describes the algorithm.

### Testing and Implementation

**Figure 2:**
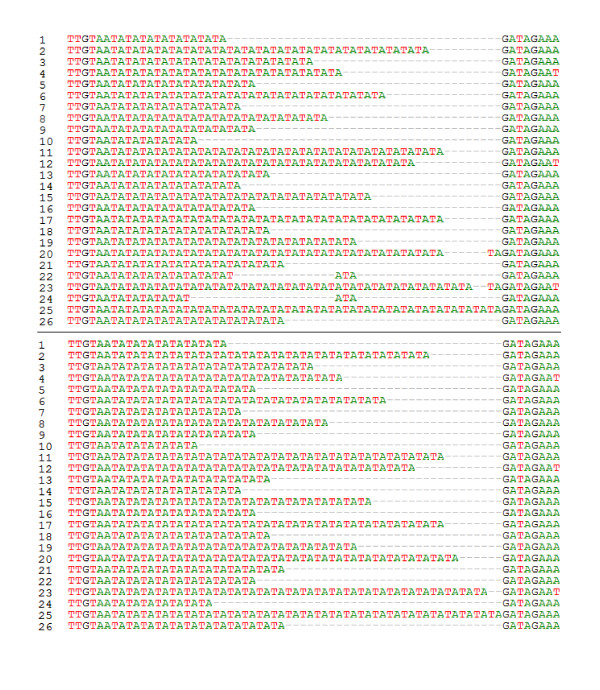
**Part of the case A sequence which contains the SSR region before (A) and after (B) applying the algorithm**.

**Figure 3:**
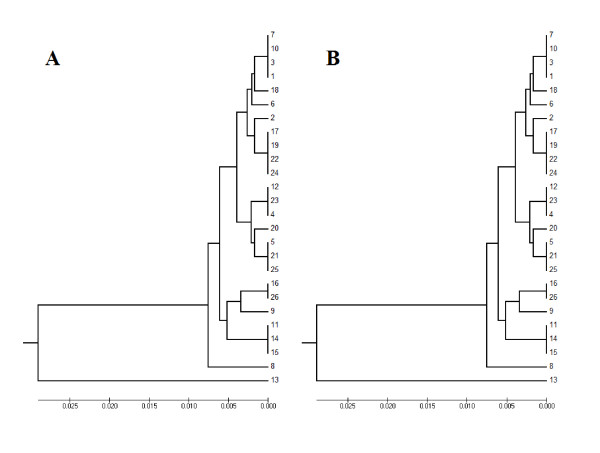
**Case A phylogenic tree before (A) and after (B) applying the algorithm**.

The sequence case A contained a simple SSR with the tandem TA, which represents 15.4% of the whole sequence. After applying the alignment in the MEGA 4 alignment and our modifications, one major difference was shown clearly in the gap sites in some sequences (Figure 2). However, these differences did not reveal variations in the phylogenic tree before and after applying the new algorithm, and the whole sequence length equals 351 bp in both cases (Figure 3).

**Figure 4:**
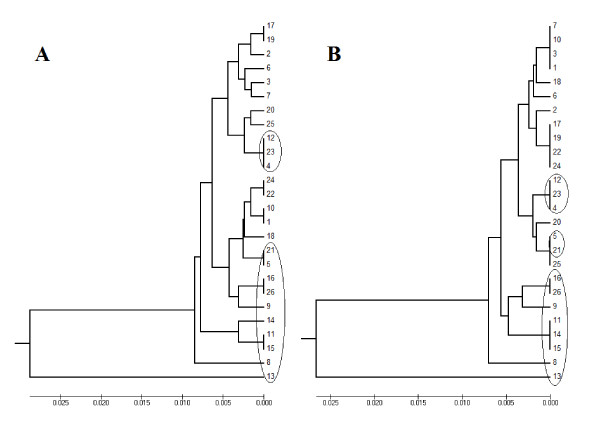
**Case B phylogenic tree before (A) and after (B) applying the algorithm**.

The sequence case B contained a compound SSR with the tandem TA and CA, which represents 25.2% of the whole sequence. The length was increased from 397 bp to 413 bp after applying the new algorithm. However, the phylogenic trees indicated that 50% of the samples showed a similar cluster before and after the new algorithm being applied (Figure 4).

**Figure 5:**
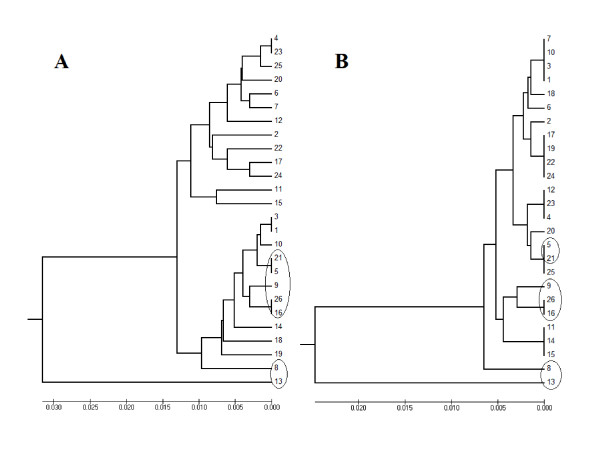
**Case C phylogenic tree before (A) and after (B) applying the algorithm**.

The sequence case C contained a compound SSR of TA, CA, and CG tandem repeats representing 35% of the whole sequence. Applying our new algorithm for case C increased the length of the sequence from 457 bp to 478 bp. However, the comparison of the phylogenic trees before and after applying the new algorithm showed that only seven samples, 26.9% of the whole sequence, clustered similarly (Figure 5).

**Figure 6:**
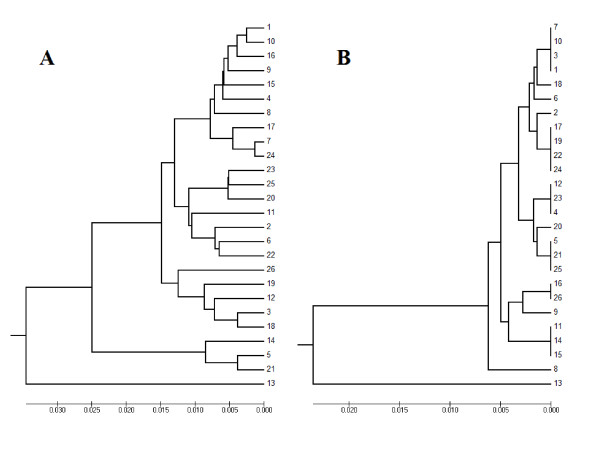
**Case D phylogenic tree before (A) and after (B) applying the algorithm**.

The sequence case D contained compound SSR (TA, CA, CG, and TG). The length of this tandem repeats represents 38% of the whole sequence. The whole sequence length was changed after the new algorithm was applied from 479 bp to 539 bp. The cluster analysis resulted in completely different phylogenic trees before and after applying the new algorithm (Figure 6).

**Figure 7:**
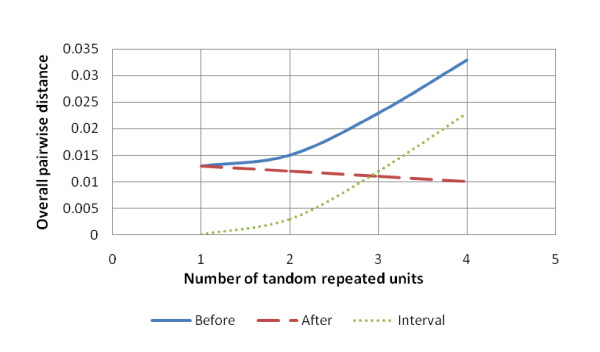
**The overall pairwise distance differences in cases A, B, C and D**.

The overall pairwise value (PV) for cases A, B, C, and D before applying the new algorithm indicated that these values were increased whenever the sequence contained more repeated units (Figure 7). In contrast, the PV was decreased after the new algorithm was applied to the same sequences. Applying the new algorithm showed a more stable distance by preventing the overlaps between different linages, although it has a slight decrease, which may be attributed to the additional aligned repeated unit, The additional units increased the SSR length giving more similarity because it does not contain overlaps or mismatches and the only difference between alleles is the opening gap position. The interval values between the two PV (before and after applying the new algorithm) were increased for the cases A, B, C, and D, indicating that the general alignment methods revealed more genetic distance.

**Figure 8:**
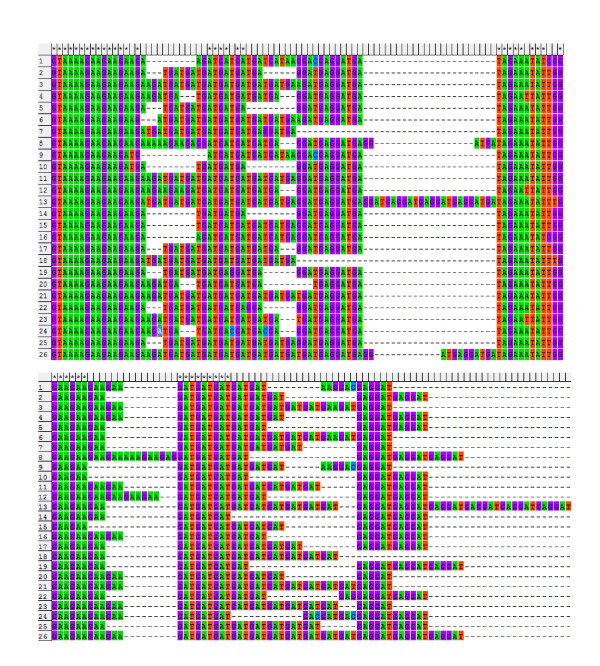
**A comparison between two alignments of the sequence of case A by using MEGA4 software (A) and the new software prepared in this paper (B)**.

**Figure 9:**
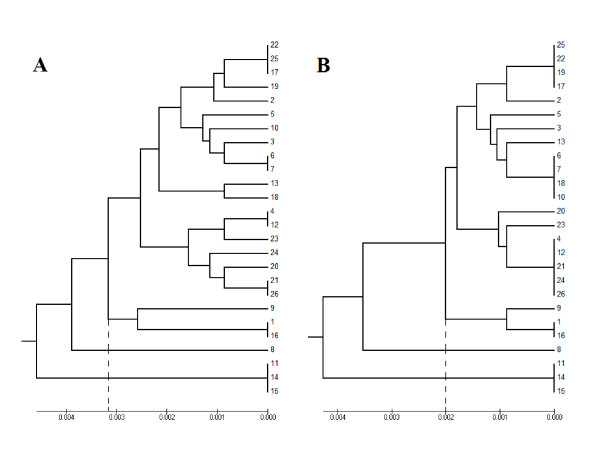
**Case E phylogenic tree before (A) and after (B) applying the algorithm**.

Case E showed a compound-imperfect SSR repeat with the tandems GAA, GAT, and GAGGAT respectively. This imperfect SSR represents 9.4% of the sequence tested in case E. The alignment process showed clear differences before and after the SSR region was treated with the new algorithm (Figure 8). Despite the small percentage of this SSR in the whole sequence in case E, the phylogenic trees showed that the genetic distance of the most 24 related sequences was decreased from 0.00317 to 0.002 (Figure 9). Further, more sequences that are similar resulted in less branches.

The main limitation with the new algorithm is in determining the gap position when applied to an imperfect SSR. According to Kruglyak [17] and Bandström [18], the imperfect repeats within the SSR region reduces the occurrences of slippage, resulting in the imperfect SSR changing its tandem nature and fixing the region by prohibiting replication slippage. This is because the bases do not find their complementary bases during replication. However, the best place for the imperfect nucleotides within a compound SSR is after the slippage site (the gap) and before the sequence that follows SSR or the next repeated unit (Figure 7).

We can deduce from the last examples that (1) the new algorithm could be a powerful tool for compound SSRs, but less so for a simple SSR, (2) it increase the similarity between sequences during alignment by minimizing the overlaps between different repeated units, and (3) it might be necessary to apply it on sequences containing long and complicated SSRs.

### SSR alignment tool (SALT)

**Figure 10:**
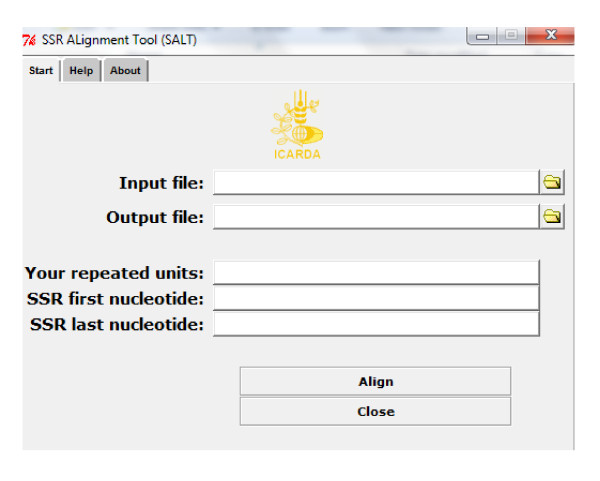
**The window of Ssr ALignment Tool (SALT)**.

SALT is a new tool for making an alignment for SSR loci using the new algorithm. It was written using the PERL programming language. Figure 10 shows the main window of the program which consists of five textboxes for the names or the directories for the input and the output files. The user should determine his tandem repeats by putting a space character between each repeated unit and the next one in the third textbox. The remaining text boxes are for identifying the first and the last nucleotide position of the SSR locus in the whole sequence. There are also four buttons, two for browsing the input and the output files, the third for making the alignment, and the last for closing the program.

The input file should be aligned sequences in fasta format or in .txt format:

1. The first line contains the number of samples, followed by any kind of separator (space or tab...) and, subsequently, the number of nucleotides.

2. Each of the next lines contains the name of the allele, followed by any kind of separator, then the sequence; thereafter press the Enter button to start the next allele.

See the additional file 2: SALT.rar. This is a compressed file containing the program and the sample data used in this research.

## Conclusions

SALT is a new tool to overcome limitations when aligning SSR loci based on the new shifting algorithm proposed in this paper. This tool is essential when aligning compound or imperfect SSRs, which contain many overlaps between repeated units, and when aligning them using the usual methods. The newly developed tool gives a better alignment estimate for such regions.

## Materials and methods

Five different sequences (Table 1) of SSR motifs obtained from a biotechnology laboratory (Genetic Resources Section, ICARDA), were used in this research. These sequences were obtained from 26 plants representing 26 alleles. The sequences were aligned using the clustalW algorithm implemented in MEGA 4 with the following default settings: gap opening penalty 15, gap extension penalty 6.66, IUB weight matrix, transition weight 0.5, and delay divergent cut-off 30 [23]. The same software drew the phylogenic tree with the UPGMA method. The PERL programming language was used to design a new algorithm for SSR alignment [24] The Tk package was used to make the graphical interface [25].

**Table 1:** Five microsatellite motifs vary in their types and lengths, representing most SSR types in the genome sequences

Case	SSR type	SSR repeat	Seq. length (bp)	SSR length (bp)	SSR (%)
A	Simple-perfect	(TA)_10_	351	54	15.4

B	Compound-perfect	(TA)_10_(CA)_16_	397	100	25.2

C	Compound-perfect	(TA)_10_(CA)_16_(CG)_14_	457	160	35

D	Compound-perfect	(TA)_10_(CA)_16_(CG)_14_(TG)_18_	479	182	38

E	Compound-imperfect	(GAA)_4_(GAT)_6_(GAGGAT)_3_	769	72	9.4

## Competing interests

The authors declare that they have no competing interests.

## Authors' contributions

AJ planned the study, wrote the PERL script, and developed the phylogenic and alignment analysis. AH was involved in the discussion of the analysis. FCO made substantial contributions towards improving the content and gave final approval to the version to be published. All the authors drafted the manuscript.

All authors read and approved the final manuscript.

## Supplementary Material

Additional file 1 An animation describes the algorithm Click here for file

Additional file 2 **A compressed file contains the program (SALT.pl) and the sample data used in this research (the folder: Sample DATA)**. (This file could be run with winrar software https://www.win-rar.com) Click here for file
